# Differentially Expressed Genes Shared by Two Distinct Cytoplasmic Male Sterility (CMS) Types of *Silene vulgaris* Suggest the Importance of Oxidative Stress in Pollen Abortion

**DOI:** 10.3390/cells9122700

**Published:** 2020-12-16

**Authors:** Manuela Krüger, Oushadee A. J. Abeyawardana, Claudia Krüger, Miloslav Juříček, Helena Štorchová

**Affiliations:** 1 Institute of Experimental Botany, Czech Academy of Sciences, Rozvojová 263, 16502 Prague, Czech Republic; kruger@ueb.cas.cz (M.K.); abeyawardana@ueb.cas.cz (O.A.J.A.); krugerc@ueb.cas.cz (C.K.); juricek@ueb.cas.cz (M.J.); 2 Department of Horticulture, Faculty of Agrobiology, Food and Natural Resources, Czech University of Life Sciences Prague, Kamýcká 129, 16500 Prague 6-Suchdol, Czech Republic

**Keywords:** cytoplasmic male sterility, *Silene vulgaris*, differential gene expression

## Abstract

Cytoplasmic male sterility (CMS), encoded by the interacting mitochondrial and nuclear genes, causes pollen abortion or non-viability. CMS is widely used in agriculture and extensively studied in crops. Much less is known about CMS in wild species. We performed a comparative transcriptomic analysis of male sterile and fertile individuals of *Silene vulgaris*, a model plant for the study of gynodioecy, to reveal the genes responsible for pollen abortion in this species. We used RNA-seq datasets previously employed for the analysis of mitochondrial and plastid transcriptomes of female and hermaphrodite flower buds, making it possible to compare the transcriptomes derived from three genomes in the same RNA specimen. We assembled de novo transcriptomes for two haplotypes of *S. vulgaris* and identified differentially expressed genes between the females and hermaphrodites, associated with stress response or pollen development. The gene for alternative oxidase was downregulated in females. The genetic pathways controlling CMS in *S. vulgaris* are similar to those in crops. The high number of the differentially expressed nuclear genes contrasts with the uniformity of organellar transcriptomes across genders, which suggests these pathways are evolutionarily conserved and that selective mechanisms may shield organellar transcription against changes in the cytoplasmic transcriptome.

## 1. Introduction

Cytoplasmic male sterility (CMS) is a special case of mitochondrial–nuclear interaction in flowering plants, leading to the inability to produce viable pollen [1,2]. CMS is caused by mitochondrial genes, which encode proteins that are harmful for mitochondrial function in the course of pollen development. CMS genes are often chimeric, composed of several pieces of other mitochondrial genes. Their expression is influenced by nuclear factors (Rf), capable of restoring male fertility [3]. Plant individuals not producing pollen develop a female (F) gender, and those with restored male fertility become hermaphrodites (H). These two genders—females and hermaphrodites—constitute the basis of the plant breeding system gynodioecy [4], the second most widespread angiosperm reproduction system after hermaphroditism [5,6].

*Silene vulgaris* has been established as a model system for the study of gynodioecy [7] and has been used in numerous population genetic studies [4,8,9,10]. The first candidate CMS gene in *S. vulgaris* (*Bobt*) was described previously [11,12], additional chimeric ORFs capable of coding for CMS were recognized in multiple completely sequenced mitochondrial genomes of *S. vulgaris* [13].

RNA-seq represents a suitable tool to study CMS, making the global comparison of mitochondrial transcriptomes between females and hermaphrodites possible. It reveals the transcripts expressed differentially between the genders, which may be involved in the metabolic processes responsible for pollen abortion. Transcriptomic studies have been conducted mainly in crops, where CMS ensures the production of hybrid seed increasing yield [14,15,16,17,18,19]. Agricultural species have passed through population bottlenecks during domestication, which reduced their genetic diversity [20,21]. For this reason, the factors driving CMS evolution in crops may not be representative of the process in wild plants. However, despite its broad occurrence in wild species, the detailed gene expression analyses of CMS are very rare, presumably because CMS candidate genes have been identified in only a few wild plants [22,23].

The identification of a CMS candidate gene [12] and non-coding RNA associated with CMS [24] in *S. vulgaris* makes it a suitable model for the investigation of transcriptomic changes accompanying CMS and pollen abortion in wild gynodioecious species. The *Bobt* gene located in the mitochondrial genome of *S. vulgaris* haplotype KRA [12] resembles other CMS genes. It is chimeric, composed of parts of *atp1* and *cox2*. In contrast, no chimeric ORF or even expressed ORF other than the genes coding for essential proteins, was found in *S. vulgaris* haplotype KOV. Instead, non-coding RNA, expressed highly in F plants, but not in H individuals, was discovered in the KOV haplotype [24]. The KRA and KOV haplotypes of *S. vulgaris* therefore carry different CMS determinants. Besides CMS-associated genes or regions, mitochondrial [12,24] and plastid [25] transcriptomes showed no significant differences between F and H plants.

Considering the general uniformity of the KRA and KOV organellar transcriptomes in *S. vulgaris*, we investigated, how much the cytoplasmic portions of the transcriptomes were different between the genders, and whether the development of male sterility shared similar genetic pathways in both the KOV and KRA haplotypes, despite their apparently distinct CMS determinants.

We utilized RNA-seq data sets previously adopted for comparative mitochondrial and plastid transcriptomic studies in two *S. vulgaris* haplotypes [12,24,25] and compared nuclear gene expression between F and H flower buds. We found general similarity of differentially expressed (DE) genes between crops and *S. vulgaris*. However, we also identified DE genes not discussed previously with regard to CMS.

## 2. Materials and Methods

### 2.1. Trimming and Quality Control of Paired-End Reads

We used RNA-seq data from total RNA extracted from flower buds of three F and three H plants of *S. vulgaris* KRA [12] and KOV [24]. The data were deposited within the NCBI Sequence Read Archive BioProject PRJNA321915. Reads from H and F plants of the KRA haplotype are saved under the accessions SRS2438489 and SRS2438490, respectively; reads from H and F plants of the KOV haplotype are saved under the accessions SRS2113743 and SRS2113744, respectively.

To obtain the reads derived from the cytoplasmic part of *S. vulgaris*, custom shell and Python scripts were used to remove all mitochondrial and plastid reads from the total paired RNA reads. In detail, all reads aligned to the mitochondrial and chloroplast/plastid genome were obtained from the corresponding BAM files, converted to FASTQ format and then, the names of these reads were saved in a list file and sorted. The Python program (filter_reads.py) was then used to compare names in the list and in the total reads file. All reads in the list were subsequently removed from the total reads files, as the resulting read files may have single (unpaired) reads, which will break pairing, these singletons were removed, too. This part was done with Python program fastqCombinePairedEnd.py and resulting paired reads saved in FASTQ format.

In the next step Rcorrector [26], which is an error correction method based on a De Bruijn graph to compactly represent all trusted k-mers in the input reads, was used, as sequencing errors in short RNA-seq reads may complicate bioinformatics analyses, e.g., alignment and assembly. Rcorrector has a higher accuracy than or comparable to existing methods, including the only other method (SEECER) designed for RNA-seq reads, and is more time and memory efficient.

After reads were error-corrected, TrimGalore, a wrapper script for quality and adapter trimming as well as quality control, was applied. Here we used TrimGalore instead of FastQC as FastQC is registering adapter contamination only if the entire sequence length (some 12 or 13 bp) is detected. The adapter trimming within Trim Galore however also removes trailing bases from reads that look like adapter sequences.

A ribosomal RNA filtering step was performed with the quality and adapter trimmed reads using SortMeRNA [27], a program tool for filtering, mapping and OTU-picking in next generation sequencing (NGS) reads. SortMeRNA takes as input a file of reads (fasta or fastq format) and one or multiple rRNA database file(s), and sorts separate rRNA from total RNA into two files specified by the user. It can provide high quality local alignments of rRNA reads against an rRNA database. We used the eukaryotic and rfam databases.

### 2.2. De Novo Assembly

The resulting reads were used for de novo assembly with Trinity v2.9.0 [28] with the in silico normalization. To remove redundancies in the assembly CD-Hit [29] was employed with an identity cutoff of 99.9%.

Finally, we used the EvidentialGene [30] tr2aacds.pl pipeline script to classify not-clustered transcripts from the Trinity assembly into valid, non-redundant coding sequences (the ‘okay’ set), and redundant, fragmented or non-coding transcripts, using the parameters-m –MINCDS = 50. We used this okay set for further analyses as this best represents the biologically real set of transcripts captured by our data.

#### Evaluation of Assemblies

For the evaluation of the different assemblies several methods were applied. First, assembly stats of the resulting assemblies were examined with the TrinityStats.pl script and are summarized in Table 1. Second, BUSCO [31], a tool to assess transcriptome completeness, based on evolutionarily-informed expectations of gene content from near-universal single-copy orthologs selected from OrthoDB v9, was used with the embryophyta_odb9 set. The results were visualized with the provided script generate_plot.py. Third, Detonate (DE novo TranscriptOme rNa-seq Assembly with or without the Truth Evaluation) RSEM-EVAL [32] was employed to further evaluate de novo transcriptome assemblies. The last method performed, was completeness and contiguity evaluation according to [33]. Completeness measures the degree to which the transcriptome is covered by the assembled contigs and is estimated by calculating the percentage of genes in the annotated gene catalog that are covered at >80% of the gene length, using an established set of previously annotated genes of the same or a closely related organism. Here, we used the gene set of *A. thaliana* (APVO SSC genes).

Contiguity measures how likely a known gene is to be assembled into a single contig covering the full length of the gene and was estimated by calculating the percentage of complete genes covered by a single contig to >80% of the gene length.

### 2.3. Estimation of Transcript Quantification and Differential Gene Expression

Transcript quantification was done with Trinity and the included scripts for downstream analyses [34]. We used the alignment based estimation method RSEM [35] with bowtie2 [36] and the alignment-free method Salmon [37] with the strand specific option—SS_lib_type RF. Followed by differential gene expression using the Bioconductor packages DESeq2 [38] and edgeR [39]. Differentially expressed features were identified with biological replicates, three for hermaphrodites and females, each. Extraction of differentially expressed genes was done within the Trinity pipeline and parameters—C 5e-2 (cutoff for FDR)—P 0 (min abs (log2(a/b)) fold change). We decided to use the statistically more rigorous FDR, improving the integrity of the set of detected DEG and minimizing the proportion of false positives below the 0.05 (5%) threshold. This value is directly related to the probability that the DEG detected are true DEG or not, while an applied log-fold change threshold (0) is not directly connected to this and does not account for the variability of the expression values. The resulting differentially expressed genes from all four methods were compared using a custom R script and visualized in a Venn diagram.

Next, blastx based homology searches (BLAST + 2.9.0) for both the KRA and KOV *S. vulgaris* de novo transcriptome assemblies against the NCBI nr protein database were performed. The cutoff E-value was set to <10^−4^, and maximum number of allowed hits was set to 10. The OmicsBox program v. 1.3.3 (BioBam Bioinformatics S.L., Valencia, Spain) was then used to annotate unigenes on the basis of gene ontology (GO) terms, InterProScan and nr database annotation. The same program was used for the visualization of the GO functional classification and the distribution of the gene functions.

To identify significantly enriched GO terms Fisher’s exact test and Gene Set Enrichment Test of OmicsBox based on an FDR-value cutoff of 0.05 were implemented. To summarize pathway information, the KEGG (Kyoto Encyclopedia of Genes and Genomes) in OmicsBox and Automatic Annotation Server (KAAS) were used to perform pathway annotation. Pathways with a *p*-value ≤ 0.05 were considered significantly enriched.

### 2.4. Reciprocal Blast Search

For the prediction of putative orthologs between *S. vulgaris* and *A. thaliana*, a reciprocal blast search was used. First, a local Blast protein database for the *S. vulgaris* translated genes was created. This database was searched for hits with the genes of the *A. thaliana* TAIR10 genome (www.arabidopsis.org). Then a list of the hits was created and these nucleotide sequences selected from the assembly of *S. vulgaris* using the script faSomeRecords (webpage is github.com/ENCODE-DCC/kentUtils/tree /master/src/utils/faSomeRecords). This reduced dataset was blasted in a second step against the database of *A. thaliana* TAIR10 proteins. Both blastx results were compared for the best hit and shared pairs picked with the python script recblast.py as best reciprocal pair and putative orthologs. The same script was applied for the search of orthologs between the *S. vulgaris* haplotypes KOV and KRA, except blastn was used instead of blastx.

### 2.5. Estimation of Gene Expression

#### 2.5.1. Plant Material

Leaf and flower bud samples were taken from 30 individuals (15 F and 15 H) with the haplotypes KRA or KOV of *S. vulgaris*. Three F and three H plants were taken from each of the parental populations KRA and KOV. To make the range of nuclear-cytoplasmic interactions as broad as possible, we also included three F and three H plants from each of the reciprocal advanced backcrosses between the KRA and KOV plants, which carried the KRA mitochondria in the KOV nuclear background and vice versa. The selection was supplemented by six plants (F1 generation) from the reciprocal crosses between KRA and KOV plants. F plants were used as mothers and H plants as pollen donors in each cross.

The plants were cultivated in the Institute of Experimental Botany (IEB) greenhouse under supplemental lighting (16/8 h light/dark) in pots filled with perlite, vermiculite, and coconut coir (1:1:1) and fertilized regularly. Three F and three H plants were sampled per cross, and plant material was frozen in liquid nitrogen and stored at −80 °C until RNA extraction.

#### 2.5.2. RNA Extraction, Reverse Transcription and qRT PCR

Total RNA was extracted using the RNeasy Plant Mini Kit (Qiagen, Hilden, Germany), followed by treatment with TURBO DNase (TURBO DNA-free™ Kit, Invitrogen, Vilnius, Lithuania) to eliminate DNA contaminations. 500 ng of RNA were used to synthesize complementary DNA (cDNA) using random hexamer primers of the Transcriptor High Fidelity cDNA Synthesis Kit (Roche Applied Science, Mannheim, Germany), as described by [24].

Quantitative PCR was performed using the LightCycler 480 SYBR Green I Master Kit and the LightCycler 480 instrument (Roche Applied Science). The reaction mixture contained 5.0 µL 2× MasterMix, primers in specific concentrations (Table S1), and 2.5 µL of 20× diluted first-strand cDNA in a total volume of 10 µL. The LightCycler was programmed with an initial denaturation for 5 min at 95 °C followed by 50 cycles of 10 s at 95 °C, 10 s at 58–60 °C (based on primers), and 15 s at 72 °C. PCR efficiencies were estimated from calibration curves generated from serial dilution of cDNAs. Three technical replicates per each specimen were measured. A calibrator (the same cDNA sample present in all plates) was used to correct for run-to-run variation. Expression values were normalized against the reference gene encoding actin (*SvAC*T), as its stable expression in *S. vulgaris* was reported by [40].

#### 2.5.3. Statistical Evaluation

The relative expression values were plotted in graphs and the outliers (zero to two in each dataset) were identified, if they were higher than twice the interquartile range (the difference between the first and third quartile). A one-way ANOVA followed by Tukey’s test as implemented in PAST v.3.14 (PAleontological STatistics) was applied on each gene expression data set.

## 3. Results and Discussion

### 3.1. Transcriptome Assembly, Reduction of Duplicates

We used Illumina raw reads from the RNA-seq data sets obtained from three female and three hermaphrodite flower buds of *S. vulgaris* haplotype KRA and haplotype KOV deposited under the Short Read Archive accession number PRJNA321915. As this data set was meant to analyze the organellar transcriptomes, which often do not possess polyA tails, polyA RNA selection was not performed [41]. Instead, rRNA was eliminated by the Ribo-Zero technique prior to cDNA library construction. To assemble the nuclear-encoded transcriptomes, we first removed all reads mapping to the organellar genomes. Then we eliminated reads derived from rRNAs, which survived rRNA elimination step before cDNA library preparation. The number of reads after the filtering steps dropped to 41% and 71% (Table 1). The decrease was higher in the KRA dataset, which might have been caused by the different read length, distinct efficiency of rRNA elimination before the library construction, or different proportion of organellar reads.

To optimize trimming, we performed de novo assembly with Trinity under various minimum length of trimmed reads, and compared the resulting assemblies based on the Detonate, BUSCO, contiguity, and completeness scores. We selected the assembly constructed from the reads with a minimum length of 146 nt (k-mer 32) for the KRA cytoplasmic transcriptome (Figure S1) and the reads with a minimum length of 65 nt (k-mer 26) for the KOV cytoplasmic transcriptome (Figure S2).

**Figure 1:**
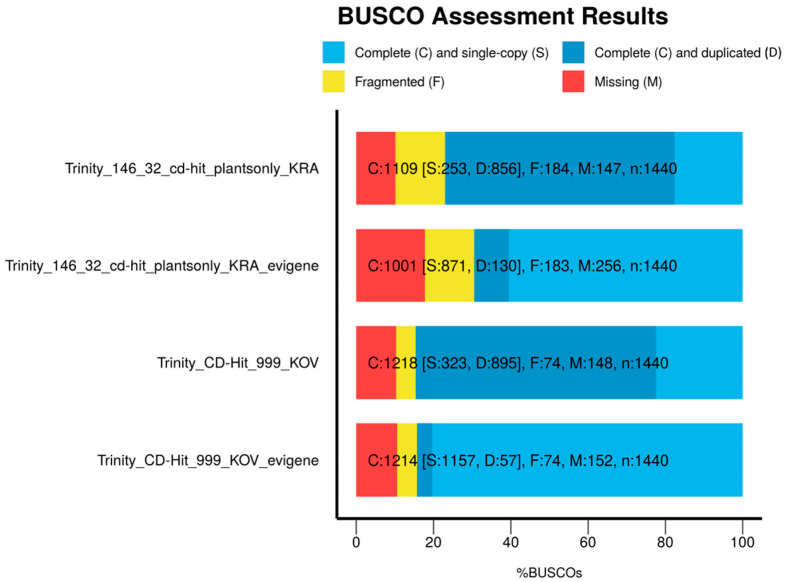
Reduction of the duplicated transcripts in the transcriptome assemblies KRA (Trinity-146_32_cd-hit-plantsonly_KRA_evigene) and KOV (Trinity_CD-Hit_999_KOV_evigene) treated with EvidentialGene.

To reduce the number of duplicates we used CD-Hit, but the resulting assemblies were still highly redundant as documented by BUSCO. We have therefore applied EvidentialGene which resulted in a dramatic decrease in the number of duplicates (Figure 1), as well as in the total number of assembled genes and transcripts. The reduction was accompanied by a slight loss of information documented by the lower values of completeness and contiguity (Table 2).

Genetic diversity and heterozygosity in *S. vulgaris* are high, as documented e.g., by [42]. This diversity may complicate both transcriptome assembly and read mapping. To ensure the maximum read mapping efficiency, we constructed a separate de novo transcriptome for each of the two *S. vulgaris* haplotypes KRA and KOV, and used it as a reference for read mapping. Whereas the KOV and KRA reads were mapped to their own assemblies from 81% and 74%, respectively, about 7% fewer reads were properly mapped against the opposite reference transcriptome. We have therefore continued the following analyses with the reference de novo transcriptomes constructed separately for each dataset.

### 3.2. Functional Annotation

**Figure 2:**
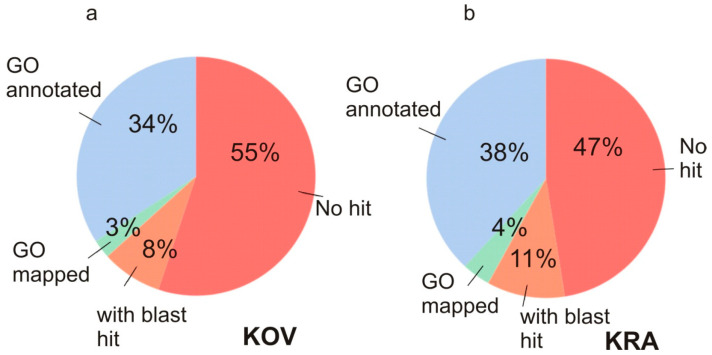
Proportion of the genes with GO annotations in (**a**) the KOV transcriptome, (**b**) in the KRA transcriptome.

The KRA and KOV transcriptomes were annotated by OmicsBox v.1.3.3 on the basis of blastx with E-value <10^−4^. 53% and 45% of Trinity ‘genes’ returned a blastx hit and 38% and 34% were annotated by B2GO from the KRA and KOV assemblies treated by EvidentialGene, respectively (Figure 2). The total number of annotated Trinity ‘genes’ was 32,022 in the KRA assembly and 22,037 in the KOV assembly. Owing to a higher number of reliably annotated genes in the KRA transcriptome, we focused primarily on the KRA assembly in the gene expression analyses.

### 3.3. Estimation of Differentially Expressed (DE) Genes

The estimation of DE genes depends on the specific method and on the selected parameter threshold. Particular caution shall be taken when the genetic material is diverse. The *S. vulgaris* plants under study were the descendants of the individuals collected in the field. These highly genetically variable wild plants contrast starkly with the genetically homogenous agricultural cultivars used for the transcriptomic studies of CMS in crops. Our original KOV and KRA datasets [12,24] were generated from the plants cultivated in the greenhouse at different time periods under slightly different conditions. The rate of morphological development of flower buds varied even among full siblings. Lower or higher expression might have been caused by slightly accelerated or delayed floral development.

**Figure 3:**
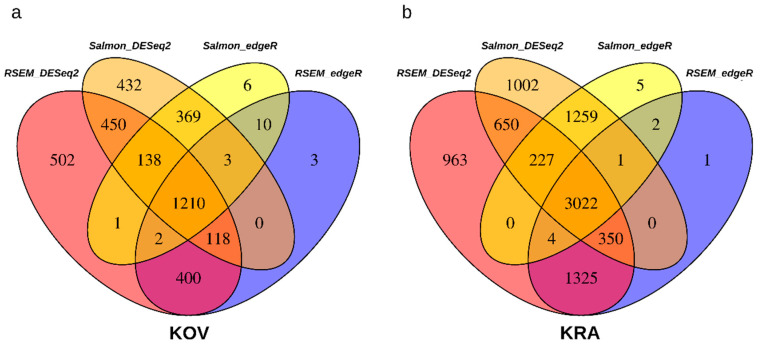
Numbers of the differentially expressed genes identified by four different bioinformatic pipelines in (**a**) the KOV transcriptome, (**b**) in the KRA transcriptome.

To identify the most reliably DE genes we applied the combination of four different methods to find DE genes between F and H flower buds (Salmon plus DESeq2, Salmon plus edgeR, RSEM plus DESeq2, RSEM plus edgeR) with FDR <0.05. We estimated 14,660 DE genes by at least one pipeline, and 1823 DE genes by all four pipelines, when we used the KRA Trinity assembly treated by CD-Hit as the reference. However, when we analyzed DE genes with the reduced KRA Trinity assembly treated by EvidentialGene as the reference, we discovered 8811 DE genes identified by at least one pipeline, and 3022 genes concurrently by all four pipelines (Figure 3b). The four methodical approaches provided much more consistent results, when the assembly was treated by EvidentialGene compared to the untreated assembly. From this reason, we used the EvidentialGene assembly for the subsequent differential expression analyses. The estimation of DE genes in the KOV transcriptomes generated similar results as the KRA transcriptomes (Figure 3a).

### 3.4. Enrichment of GO Categories

We applied Fisher’s exact test implemented in OmicsBox v.1.3.3 (FDR < 0.05) to find significantly enriched GO terms. We processed all the annotated transcripts identified as significantly downregulated or upregulated in females by all four pipelines as described above. We found 315 enriched GO categories among downregulated DE genes and 174 enriched GO categories among upregulated DE genes in the KRA transcriptomes (Table S2). The most specific GO categories downregulated in females are depicted in Figure S3. They include cell wall modification, sporopolenin biosynthesis, tapetum morphogenesis, sugar transmembrane transporter, fatty acid elongation, triglyceride biosynthesis, and cellular iron ion homeostasis. The most specific GO terms upregulated in females are shown in Figure S4, including responses to salt and oxidative stresses or wounding, leaf senescence, peroxidase activity, abscisic acid signaling pathway, or nitrate transport.

The GO enrichment analysis of the KOV transcriptomes produced similar results as the KRA transcriptomes with the genes upregulated in females, but no enriched GO terms were found in the set of downregulated genes at FDR <0.05. This result may be related to the lower number of transcripts assembled (Table 2) and annotated (Figure 2) in the KOV transcriptome, constructed from shorter reads with minimum length of 65 nt compared to its KRA counterpart, generated from reads with minimum length of 146 nt.

### 3.5. DE Genes Shared by KRA and KOV Plants

We aimed to find the genes which are activated or inhibited in female flower buds of *S. vulgaris* regardless of the mitochondrial haplotype. We applied reciprocal blast hit to identify the transcripts orthologous between KRA and KOV haplotypes. 31,081 orthologous pairs were found among 84,004 KRA (38%) and 64,549 KOV Trinity ‘genes’ (48%) in the final Evigene-treated assemblies. 21,629 (70%) orthologous pairs returned blastx hits, suggesting that the proportion of annotated genes was higher among orthologs between KRA and KOV than in the entire gene set.

We selected the orthologous pairs identified as DE genes by all four methods in both KRA and KOV transcriptomes to get a reliable set of DE genes shared between the KRA and KOV plants. There were 162 downregulated (104 with an informative blastx hit) (Table 3) and 82 (76 with an informative blastx hit) upregulated orthologs (Table 4). In addition, we compiled a more comprehensive list of 468 annotated orthologous pairs, which were recognized as DE genes by at least one of the four methods in both KRA and KOV plants (Table S3).

We also searched for DE genes specific only to one haplotype, either KOV or KRA, with not being DE in the other. However, we found no ortholog identified as a DE gene by all four (or at least three) methods in one transcriptome, which was not at the same time recognized as DE by at least one method in the other transcriptome. This result does not mean that such genes do not exist. They may be present, but they did not meet our criteria. The KOV transcriptomic assembly contained less transcripts with shorter average length (Table 2), which might have complicated the correct ortholog identification, particularly in less expressed genes with a higher risk of transcript misassembly. We are now working on a comprehensive comparison of the KRA and KOV haplotypes with transcriptomes sampled and sequenced at the same time, which also includes backcrossed plants with exchanged mitochondria, harboring KOV mitochondria in the KRA nuclear background and vice versa. This study will reveal the DE genes specific for the particular CMS type, if they exist.

Our results document numerous genes differentially expressed between F and H flower buds, which were shared by the KRA and KOV haplotypes of *S. vulgaris* carrying distinct CMS types. We describe the putative functions of selected significantly DE genes in the following paragraphs.

### 3.6. Genes Involved in Male Functions Were Downregulated in S. vulgaris Female Flower Buds

Numerous DE genes downregulated in *S. vulgaris* females were associated with male structures—pollen or anthers (Table 3). For example, the *SMALL AUXIN UP RNA (SAUR) 63* gene subfamily represented by DN45544_c4_g1 regulates gibberellin-dependent stamen filament elongation in *A. thaliana* [43].

Tapetum, the innermost layer of anther ensuring the nutrition of developing microspores and pollen grains [44], is particularly important in pollen development. The abnormal development of tapetal tissue may result in the degradation of microspores [45]. The GO terms ‘sporopolenin biosynthesis’ or ‘tapetum morphogenesis’ were enriched among the downregulated genes. The *NODULIN INTRINSIC PROTEIN 7*;*1*(*NIP7*;*1*) gene (DN49199_c0_g1) codes for a channel supplying boron necessary for pollen cell wall synthesis, which is expressed in the tapetum of *A. thaliana* [46]. Another downregulated transcript (DN57560_c0_g2) is homologous to *BARELY ANY MERISTEM (BAM2*) encoding putative leucine-rich repeat receptor-like kinase, which controls tapetal cells’ development in *A. thaliana* [47]. The MYB transcription factor ABORTED MICROSPORES (AMS) (DN59645_c1_g1) regulates tapetal cell development and microspore formation and is associated with genic male sterility in watermelon [48].

In addition to the genes with known function, a homolog of *serendip2*, (DN62179_c1_g1) specific to the male gender in *Silene latifolia* [49] was also inhibited in *S. vulgaris* females. This gene has not been found in any other plant species.

The downregulation of the genes involved in lipid and carbohydrate metabolism may harm the biosynthesis of the pollen cell wall and its outer layer exine, produced by the tapetum [50,51]. We observed the inhibition of several genes related to fatty acid synthesis, wax and cutin formation in *S. vulgaris* females (two transcripts coding for pectinesterases (DN44106_c0_g1, DN69711_c0_g1), the transcript for UDP-arabinopyranose mutase (DN59301_c0_g4) involved in cell wall modifications [52]). Genes with similar functions were also inhibited in male sterile individuals e.g., in radish and other crops [14,15,16,17,18,19,53]. In contrast, the transcripts coding for catabolic enzymes lipases and esterase (DN57332_c1_g2, DN61032_c0_g1), or caleosin (DN54044_c1_g1), functioning in degradation of storage lipids, were upregulated in females (Table 4).

Besides the roles played in cell wall biosynthesis and modifications, carbohydrate metabolism supplies the energy necessary in the course of pollen development. The transcripts coding for the enzymes hydrolyzing polysaccharides such as alpha-amylase (DN35498_c0_g1) (Table 4) or beta-amylase1 (DN67781_c0_g1) (Table S2) were upregulated in *S. vulgaris* females.

### 3.7. Pollen Abortion Is Associated with Oxidative Stress

**Figure 4:**
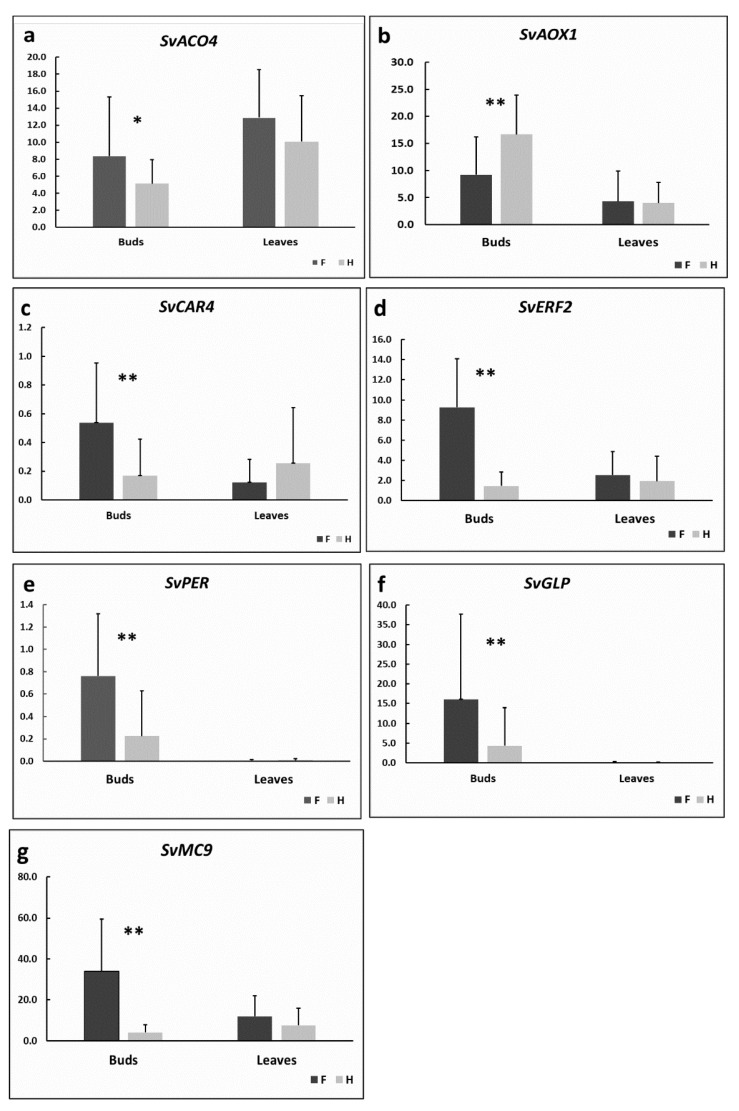
Relative expression estimates of selected DE genes between F and H plants of *S. vulgaris*. The gene coding for actin was used as a reference. Fifteen individuals of each gender were analyzed. Significant differences, as estimated by ANOVA (*p* < 0.05), are marked by two asterisks. The result with (0.05 < *p* < 0.07) is marked with a single asterisk. (**a**). *SvACO4*, (**b**). *SvAOX1*, (**c**). *SvCAR4*, (**d**). *SvERF2*, (**e**). *SvPER*, (**f**). *SvGLP*, (**g**). *SvMC9*.

Many oxidative stress-induced genes (e.g., encoding ROS scavenging enzymes such as peroxidases) were strongly upregulated in females of *S. vulgaris*, which suggests the impact by oxidative stress. The example is the transcript DN56053_c0_g3 coding for peroxidase (PER). The RT qPCR estimation of the *SvPER* transcript levels confirmed its expression in flower buds of both genders, but not in leaves (Figure 4e). This finding may be explained by the elevated production of ROS with subsequent induction of peroxidases in the course of the development of floral organs, but not during the leaf development. ROS in non-green tissues are primarily produced due to excessive electrons generated in mitochondria [45,54]. The increased ROS production in flower buds can be associated with the elevated mitochondrial respiration needed to satisfy the high energy demands of pollen and ovule development.

The impairment of mitochondrial respiration in female plants caused by the activity of mitochondrial CMS genes may lead to accelerated ROS accumulation resulting in pollen abortion [1,2]. The higher ROS concentrations in male sterile than in fertile anthers were reported e.g., by [55] in their study of CMS-D8 in cotton. Similar results were obtained by [56], who described enhanced ROS production interfering with pollen development in CMS-WA in rice.

The signaling function of some ROS ensuring the proper timing of programmed cell death in the course of standard pollen development should also be considered [57]. Transcripts encoding cell death-related proteins were upregulated in females, for example the gene for metacaspase 9 (MC9), a protease involved in cell death regulation [58] (DN35718_c0_g2). *SvMC9* upregulation in F plants was confirmed by RT qPCR (Figure 4).

The response to the oxidative stress is tightly associated with phytohormones. ABA and ethylene among the phytohormones are involved in plants stress responses. The genes responsible for ethylene production or signaling (the transcript DN48287_c2_g1 encoding 1-aminocyclopropane-1-carboxylate oxidase (ACO4), the rate-limiting enzyme in ethylene biosynthesis; the transcript DN62269_c0_g1 coding for ETHYLENE-RESPONSIVE TRANSCRIPTION FACTOR 2 (ERF2), inducible by abiotic stresses), or ABA signaling (the transcript DN48556_c0_g2 encoding C2-DOMAIN ABA-RELATED 4 (CAR4) facilitating ABA signaling [59]) were upregulated in female flower buds of *S. vulgaris*, most likely induced by oxidative stress. RT qPCR confirmed their increased expression in F flower buds, whereas no differences between the genders were measured in leaves (Figure 4a,c,d). Elevated concentrations of ABA in male sterile flowers of *Brassica napus* were previously reported by [60].

We also observed the high upregulation of several transcripts encoding germin-like proteins (GLPs) in female flower buds. These proteins are induced by a plethora of biotic and abiotic stresses [61], but their role in CMS or pollen abortion has not been studied yet. The *SvGLP* gene exhibited very low or zero expression in leaves, as detected by RT qPCR (Figure 4f), which may be associated with lower ROS concentrations in leaves than in flowers.

### 3.8. AOX1 Gene Was Downregulated in Female Flower Buds in S. vulgaris

Alternative oxidase 1 (AOX1) is the enzyme involved in the alternative route of transferring electrons to oxygen, which bypasses cytochrome c oxidases and ATP production. AOX1 prevents the accumulation of ROS [62] and is induced by blocking mitochondrial respiration or by environmental conditions [63,64]. AOX1 is considered to be a marker of retrograde signaling directed from mitochondria to the nucleus [65].

In contrast with most stress-related genes, the transcript DN56446_c0_g1 encoding AOX1 was higher in H than in F flower buds. The lowest *AOX1* expression was detected by RT qPCR in the leaves of both genders (Figure 4b). The peroxidase gene *SvPER* or stress-response gene encoding germin-like protein *SvGLP* also exhibited very low or zero expression in leaves, which may be associated with lower ROS concentrations in leaves than in flowers. However, unlike *SvPER* or *SvGLP* genes, *AOX1* transcript levels were lower in F flower buds compared with H flower buds. This result contradicts the study of CMS of [66] in cotton, that found higher level of *AOX1* in F flowers. The authors of the recent study [67] imported RNA associated with CMS to mitochondria of *A. thaliana* and described increased or decreased levels of *AOX1* depending on the time since RNA import, developmental stage, or light conditions.

These reports suggest that the control of *AOX1* expression during CMS is complex. It may depend on the developmental stage of flower, the CMS type, or the species. The decrease of *AOX1* expression in F flower buds in two distinct CMS types in *S. vulgaris* may trigger ROS accumulation, which launches untimely degradation of the tapetum leading finally to pollen abortion and male sterility. As AOX1 participates in retrograde signaling, our results indicate a prominent role of this process in CMS, in line with [68,69].

### 3.9. Differences between Nuclear-Encoded Transcriptomes of F and H Flower Buds of S. vulgaris Contrast with the Uniformity of Their Organellar Transcriptomes

Transcripts coding for proteins targeted to plastids or mitochondria were frequently present among DE genes between F and H flower buds. They included several transcripts involved in lipid metabolism targeted to plastids, as well as the transcripts responsible for plastoquinone synthesis (DN50701_c1_g1, DN64967_c1_g1). DE transcripts involved e.g., in amino acid biosynthesis (DN70324_c1_g1), or oxidation-reduction processes (DN64912_c1_g4) were targeted to mitochondria. The oxidation resistance protein 1 (DN65942_c2_g2), a mitochondrial protein induced by oxidative stress [70], was downregulated in F plants, similarly to *AOX1*.

We analyzed the cytoplasmic portions of the RNA-seq data sets, from which organellar transcriptomes were mapped previously. We might have therefore directly compared the transcriptomes from three compartments—mitochondrial, plastid, and cytoplasmic—constructed from the same RNA samples. The mitochondrial transcriptomes exhibited only very few differences in coverage or editing between F and H [12,24], the plastid transcriptomes were completely identical between F and H [25]. Our observations contradict some studies, e.g., [19], which described numerous mitochondrial DE genes between male sterile and male fertile individuals. This discrepancy may be sometimes explained by experimental artifacts. Liu et al. [19] used polyA enrichment for the construction of cDNA library, but this method cannot detect organellar transcripts that lack poly A [41] and may lead to the underestimation of gene coverage.

Considering thousands of nuclear-encoded genes DE between F and H flower buds of *S. vulgaris*, including many genes coding for plastid and mitochondria targeted proteins, the uniformity of organellar transcriptomes of *S. vulgaris* is surprising. Niazi et al. [67] described the stability of the mitochondrial transcriptome of *A. thaliana*, which was capable of buffering the changes associated with knocking down the selected mitochondrial genes. Accordingly, our results support the existence of mechanisms balancing the steady transcription profiles of organellar genes of *S. vulgaris* even under conditions leading to pollen abortion.

## 4. Conclusions

We revealed numerous nuclear-encoded DE genes between F and H flower buds of the gynodioecious species *S. vulgaris.* Our findings contrast with near-zero differences in gene expression in mitochondria or plastids of *S. vulgaris*, when estimated in the same RNA samples, suggesting the existence of a mechanism shielding organellar transcription against the changes in the cytoplasmic transcriptome.

We observed a general similarity between the genetic pathways controlling CMS in crops and the wild gynodioecious species *S. vulgaris*. However, we also discovered some DE genes, not reported or discussed in previous CMS studies, e.g., *serendip* or the genes coding for germin-like proteins. The *AOX1* transcripts were reduced in F flower buds, suggesting that *AOX1* downregulation may contribute to ROS accumulation, which ultimately results in pollen abortion.

## Figures and Tables

**Table 1:** Number of reads used for the construction of the KRA and KOV transcriptomes. Initial numbers of raw reads are compared with the final numbers after quality filtration and the removal of the reads derived from rRNA and organellar transcripts.

KRA	Raw Reads	Final Number of Reads
Sum of read pairs	232,291,523	96,244,096
Average number of read pairs per sample	38,715,254	16,040,683
KOV		
Sum of read pairs	199,896,220	142,034,627
Average number of read pairs per sample	33,316,036	23,672,438

**Table 2:** Comparison of the KOV and KRA assemblies of *S. vulgaris* generated by Trinity and reduced by CD Hit and EvidentialGene.

Assembly	N50	Percent GC	Total Trinity ‘Transcripts’	Total Trinity ‘Genes’	Transcripts’ per ‘Genes’	Average Contig	Median Contig Length	Total Assembled Bases	Contiguity	Completeness	Detonate Score
KRA											
Trinity_146_32	1477	39.51	485,454	217,940	2.227466275	943	598	457,681,231			
Trinity_146_32_cdhit	1486	39.4	459,631	198,260	2.318324422	960	621	441,163,523	0.827	0.905	−39,167,557,352
Trinity_146_32_evigene	1387	39.71	104,013	84,004	1.238191039	951	662	98,923,714	0.783	0.85	−39,482,140,762
KOV											
Trinity_65	1280	39.76	322,441	181,371	1.777797994	774	437	249,686,710			
Trinity_65_cdhit	1268	39.7	307,905	170,448	1.806445368	779	449	239,849,174	0.868	0.909	−23,349,399,191
Trinity_65_evigene	1300	40.1	72,262	64,549	1.11949062	822	470	59,426,744	0.863	0.897	−28,051,238,486

**Table 3:** Orthologous genes between the KRA and KOV haplotypes downregulated in F flower buds.

KOV	KRA	Annotation	Process or Function
DN20956_c0_g1	DN25290_c0_g1	aquaporin TIP1-3	abiotic stress response
DN8218_c1_g1	DN50548_c0_g1	BURP domain protein RD22	abiotic stress response
DN11001_c0_g2	DN58656_c0_g2	SPX domain-containing protein 3	abiotic stress response
DN117378_c0_g1	DN52115_c3_g1	ABC transporter G family member 14	abiotic stress response, transport of cytokinins
DN117904_c0_g1	DN57560_c0_g2	leucine-rich repeat receptor-like kinase BAM2	anther and pollen development
DN17312_c0_g1	DN45544_c4_g1	auxin-responsive protein SAUR64	anther development
DN17927_c0_g2	DN49199_c0_g1	aquaporin NIP7-1	anther development
DN19580_c0_g2	DN42368_c0_g1	scopoletin glucosyltransferase	biotic stress response
DN29923_c0_g1	DN49507_c0_g1	glucan endo-1,3-beta-glucosidase 12	biotic stress response
DN13581_c1_g1	DN50185_c1_g1	anthranilate N-benzoyltransferase 2	biotic stress response
DN294_c0_g1	DN63593_c0_g1	inositol-pentakisphosphate 2-kinase	biotic stress response
DN5945_c0_g1	DN64094_c0_g1	ABC transporter G family member 31	biotic stress response
DN26761_c0_g1	DN39252_c0_g1	F-box protein CPR30-like	biotic stress response, proteasome
DN49774_c0_g1	DN39675_c0_g1	F-box/LRR-repeat protein 13-like	biotic stress response, proteasome
DN15243_c0_g1	DN52671_c0_g1	jasmonate-induced protein homolog	biotic stress response, response to jasmonate
DN36891_c0_g2	DN54344_c0_g1	protein TIFY 10A-like	biotic stress response, response to jasmonate
DN5404_c2_g1	DN44106_c0_g1	pectinesterase 11	cell wall
DN3555_c0_g2	DN48381_c0_g1	xyloglucan endotransglucosylase/hydrolase	cell wall
DN75992_c1_g1	DN59301_c0_g4	UDP-arabinopyranose mutase 1	cell wall
DN6141_c0_g1	DN60757_c0_g1	cell wall RBR3	cell wall
DN12797_c0_g1	DN69711_c0_g1	Pectinesterase 2	cell wall, pectin modification
DN11562_c0_g1	DN54928_c0_g1	UDP-glucuronate:xylan alpha-glucuronosyltransferase	cell wall, sugar metabolism
DN24940_c0_g2	DN68925_c2_g1	microtubule-associated protein 70-2	cytoskelet
DN28090_c0_g1	DN23353_c0_g1	LIM domain-containing protein WLIM2b	cytoskelet
DN3731_c0_g1	DN55753_c0_g1	protein NSP-INTERACTING KINASE 1	defense against viruses
DN5587_c1_g1	DN52217_c0_g1	ribosome-inactivating protein lychnin	defense to herbivores
DN15662_c0_g1	DN54291_c1_g1	benzyl alcohol O-benzoyltransferase	floral scent production
DN1200_c0_g1	DN66313_c0_g2	benzyl alcohol O-benzoyltransferase	floral scent production
DN12658_c0_g1	DN61319_c0_g1	ent-copalyl diphosphate synthase, chloroplastic	gibberellin biosynthesis
DN10021_c0_g1	DN67192_c0_g1	3-hydroxy-3-methylglutaryl-coenzyme A reductase 3	isoprenoid biosynthesis
DN7797_c0_g3	DN58587_c0_g1	caffeoylshikimate esterase-like	lignin biosynthesis
DN84_c0_g3	DN64134_c1_g1	O-acyltransferase WSD1-like	lipid and wax metabolism, cutin synthesis
DN1640_c0_g1	DN72417_c1_g1	protein ECERIFERUM 1-like	lipid and wax metabolism, cutin synthesis
DN18997_c0_g1	DN62098_c0_g1	GDSL esterase/lipase EXL3	lipid catabolism
DN1956_c0_g1	DN48468_c3_g1	very-long-chain 3-oxoacyl-CoA reductase 1	lipid metabolism
DN12582_c0_g2	DN49044_c1_g1	putative peroxygenase 4	lipid degradation, ABA signaling
DN4033_c0_g1	DN50049_c3_g1	delta(12)-fatty-acid desaturase FAD2	lipid metabolism
DN28833_c0_g1	DN55962_c0_g1	Acyltransferase-like protein, chloroplastic	lipid and wax metabolism, cutin synthesis
DN6801_c0_g1	DN56028_c0_g2	acyl-CoA--sterol O-acyltransferase 1	lipid metabolism
DN1868_c0_g1	DN61281_c1_g1	3-oxoacyl-[acyl-carrier-protein] synthase I, chloroplastic	lipid metabolism
DN4218_c0_g4	DN61881_c0_g1	long chain acyl-CoA synthetase 7, peroxisomal	lipid metabolism
DN5733_c0_g3	DN62382_c1_g1	NADH--cytochrome b5 reductase 1	lipid metabolism
DN12960_c0_g1	DN65129_c0_g1	acyltransferase-like At1g54570, chloroplastic	lipid and wax metabolism, cutin synthesis
DN7091_c0_g1	DN70722_c3_g2	non-specific lipid-transfer protein A-like	lipid metbolism, cutin formation
DN9230_c0_g1	DN57659_c0_g1	glycerol-3-phosphate 2-O-acyltransferase 6	lipid metabolism, cutin synthesis
DN8493_c1_g4	DN58552_c1_g2	dehydrodolichyl diphosphate synthase subunit NUS1-like	lipid metabolism, glycosylation
DN4923_c0_g1	DN71674_c2_g5	dehydrodolichyl diphosphate synthase subunit NUS1-like	lipid metabolism, glycosylation
DN42303_c0_g1	DN54377_c1_g1	3-ketoacyl-CoA synthase 10	lipid metabolism, pollen development
DN325_c0_g1	DN69099_c0_g1	3-ketoacyl-CoA synthase 7-like	lipid metabolism, very long chain fatty acid
DN40822_c0_g1	DN56377_c0_g1	Adipose-regulatory protein, putative	lipid storage
DN17020_c0_g1	DN52633_c0_g1	aluminum-act. malate transporter 2	malate transport
DN140371_c0_g1	DN62179_c1_g1	serendip2, partial	male gender-associated gene
DN19368_c0_g1	DN58293_c0_g1	non-functional NADPH-dependent codeinone reductase 2	oxidation-reduction processes
DN1066_c0_g2	DN58866_c0_g1	cytochrome P450 89A2	oxidation-reduction processes
DN8357_c0_g1	DN63019_c1_g2	uncharacterized oxidoreductase At1g06690, chloroplastic	oxidation-reduction processes
DN8866_c0_g2	DN64912_c1_g4	nudix hydrolase 12, mitochondrial-like	oxidation-reduction processes
DN1426_c0_g1	DN64077_c0_g1	cell number regulator 13-like	plant growth
DN6949_c0_g2	DN64967_c1_g1	2-methyl-6-phytyl-1,4-HQ methyltransferase, chloroplastic	plastoquinon synthesis
DN3074_c0_g2	DN41000_c0_g1	plant self-incompatibility protein S1 family	pollen development
DN5323_c0_g2	DN43413_c0_g1	Plant self-incompatibility S1	pollen development
DN881_c0_g1	DN70592_c0_g1	polygalacturonase QRT3-like	Pollen development
DN10605_c0_g1	DN62065_c1_g1	serine/threonine-protein phosphatase PP1	protein dephosphorylation
DN5982_c0_g1	DN46942_c0_g5	peptidyl-prolyl cis-trans isomerase	protein folding
DN3850_c0_g1	DN67053_c0_g2	beta-1,3-galactosyltransferase 8	protein glycosylation
DN24059_c0_g1	DN43126_c0_g1	ervatamin-B isoform X2	proteolysis
DN96143_c0_g2	DN50973_c0_g1	F-box/kelch-repeat protein At3g23880	proteolysis
DN10994_c0_g2	DN55415_c0_g4	probable aspartic protease At2g35615	proteolysis
DN43449_c0_g1	DN66566_c0_g1	F-box/kelch-repeat protein At3g23880	proteolysis
DN1779_c0_g1	DN56281_c3_g1	HIG_1_N domain-containing protein	respiration in mitochondria
DN8887_c0_g1	DN56446_c0_g1	alternative oxidase1, mitochondrial	respiration in mitochondria
DN6741_c1_g2	DN67021_c1_g1	aldehyde dehydrogenase family 2 member C4	response to biotic stress, redox processes
DN15427_c0_g1	DN70278_c1_g1	aldehyde dehydrogenase family 3 member F1	response to osmotic stress
DN2886_c0_g2	DN65942_c2_g2	oxidation resistance protein 1	response to oxidative stress
DN960_c0_g2	DN54803_c0_g2	plastid-lipid-associated protein 3, chloroplastic	response to stress, jasmonate synthesis
DN79910_c0_g1	DN59524_c0_g2	protein SUPPRESSOR OF MAX2 1	response to strigolactones
DN3860_c0_g1	DN39636_c0_g2	retroelement pol polyprotein-like	retroelement
DN22296_c0_g2	DN43632_c0_g1	RNA-binding protein pno1-like	RNA binding
DN100112_c0_g1	DN53301_c0_g3	annexin-like protein RJ4	abiotic stress response
DN11360_c0_g1	DN69045_c0_g1	cation/H(+) antiporter 20	abiotic stress response, stomata opening
DN25736_c0_g1	DN48226_c0_g1	mitogen-activated protein KKK 1	stress response
DN88774_c0_g1	DN50043_c0_g2	Bet beta-fructofuranosidase	sugar metabolism
DN49169_c0_g2	DN54840_c0_g2	rhamnogalacturonan I rhamnosyltransferase	sugar metabolism
DN664_c0_g1	DN73158_c6_g2	protein TSS	suppression of meristem proliferation
DN14694_c0_g1	DN52676_c1_g1	zinc finger protein ZAT4	TF, abiotic stress response
DN64000_c0_g1	DN50222_c0_g3	transcription factor MYB26	TF, anther development
DN31894_c0_g1	DN60959_c0_g2	protein FAR1-RELATED SEQUENCE 5	TF, development
DN26183_c0_g1	DN53805_c0_g3	bZIP transcription factor 11	TF, hypo-osmolarity stress response
DN29604_c0_g1	DN47072_c0_g5	NAC transcription factor 25	TF, pollen development
DN2940_c0_g1	DN63243_c0_g1	transcription factor MYB97	TF, pollen development
DN1229_c0_g1	DN59645_c1_g1	transcription factor ABORTED MICROSPORES	TF, pollen development
DN2456_c0_g1	DN62952_c0_g1	BEL1-like homeodomain protein 11	TF, positive regulation of plastid development
DN14971_c1_g2	DN52803_c0_g1	transcription factor MYB41	TF, salt stress response
DN5746_c0_g1	DN50022_c0_g1	transcription factor MYB44	TF, stress response
DN11354_c3_g1	DN56745_c0_g3	ubiquitin-60S ribosomal protein L40	translation
DN4532_c0_g2	DN46765_c0_g1	eukaryotic translation initiation factor 3 subunit G	translation initiation
DN8567_c0_g1	DN58593_c1_g3	40S ribosomal protein S27-2	translation, abiotic stress response
DN263_c0_g1	DN57992_c0_g2	Ctr copper transporter	transport of copper
DN22803_c0_g1	DN49878_c0_g1	fe(2+) transport protein 1	transport of iron
DN34096_c0_g1	DN63070_c0_g1	metal-nicotianamine transporter YSL7	transport of metals
DN8241_c1_g1	DN73009_c4_g1	ABC transporter-like	transport of phytohormones
DN6894_c0_g1	DN63590_c0_g1	purine permease 5	transport of purines
DN16716_c1_g1	DN55273_c0_g1	equilibrative nucleotide transporter 3	transport of nucleotides, cytokinins
DN4674_c0_g1	DN50701_c1_g1	homogentisate solanesyltransferase, chloroplastic	ubiquinone synthesis
DN10150_c0_g1	DN63897_c0_g1	methyltransferase At1g78140, chloroplastic	ubiquinone synthesis

TF—transcription factor; green color—proteins likely targeted to plastids; blue color—proteins likely targeted to mitochondria.

**Table 4:** Orthologous genes between the KRA and KOV haplotypes upregulated in F flower buds.

KOV	KRA	Annotation	Process or Function
KOV_DN16097_c0_g1	KRA_DN47101_c0_g1	caffeine synthase 1-like	alkaloid metabolism
KOV_DN17890_c0_g1	KRA_DN67656_c0_g2	CBL-interacting serine/threonine-protein kinase 20	ABA response
KOV_DN2739_c0_g1	KRA_DN70411_c3_g1	ABC transporter G family member 25	ABA transport
KOV_DN93524_c0_g1	KRA_DN48556_c0_g2	protein C2-DOMAIN ABA-RELATED 4-like	ABA signaling
KOV_DN84273_c0_g1	KRA_DN67096_c1_g1	UPF0496 protein At1g20180-like	abiotic stress response
KOV_DN1869_c0_g1	KRA_DN49644_c0_g1	homocysteine S-methyltransferase 3	amino acid metabolism
KOV_DN30024_c0_g1	KRA_DN52586_c0_g1	cystinosin homolog	amino acid transport
KOV_DN17245_c0_g1	KRA_DN59599_c0_g1	amino acid permease 6-like	amino acid transport, nutrition
KOV_DN2748_c0_g1	KRA_DN70324_c1_g1	gamma aminobutyrate transaminase 1, mitochondrial	amino-acid metabolism
KOV_DN1890_c0_g4	KRA_DN56949_c0_g1	branched-chain-amino-acid aminotransferase 2, chloroplastic	amino-acid synthesis
KOV_DN60253_c0_g1	KRA_DN60612_c1_g1	protein PIN-LIKES 7-like	auxin response
KOV_DN51736_c2_g4	KRA_DN41168_c0_g1	NDR1/HIN1-like protein 1	biotic stress response
KOV_DN4107_c0_g1	KRA_DN47018_c0_g1	isoflavone 3′-hydroxylase-like	biotic stress response
KOV_DN3773_c0_g1	KRA_DN52943_c0_g3	UPF0496 protein At4g34320-like	biotic stress response
KOV_DN1836_c0_g3	KRA_DN55452_c0_g3	acidic mammalian chitinase-like	biotic stress response
KOV_DN4184_c0_g2	KRA_DN59528_c0_g4	protein ENHANCED PSEUDOMONAS SUSCEPTIBILTY 1	biotic stress response
KOV_DN1416_c0_g1	KRA_DN64933_c1_g2	probable serine/threonine-protein kinase PBL19	biotic stress response
KOV_DN4164_c0_g1	KRA_DN54390_c0_g2	protein NRT1/PTR FAMILY 5.2-like	biotic stress response, peptide transport
KOV_DN14790_c0_g1	KRA_DN69798_c2_g1	cyclic nucleotide-gated ion channel 2-like	biotic stress response, programmed cell death
KOV_DN9654_c0_g1	KRA_DN55792_c0_g1	formate dehydrogenase, mitochondrial	cell death, biotic stress response
KOV_DN6043_c0_g1	KRA_DN35718_c0_g2	metacaspase 9-like	cell death
KOV_DN18472_c0_g1	KRA_DN61497_c0_g1	protein NEN1	cell death, nuclease
KOV_DN16884_c0_g1	KRA_DN59879_c0_g1	endonuclease 1	cell death, senescence
KOV_DN1688_c0_g1	KRA_DN51302_c1_g2	vignain-like	programmed cell death, proteolysis
KOV_DN3555_c0_g1	KRA_DN51950_c0_g1	probable xyloglucan endotransglucosylase/hydrolase30	cell wall
KOV_DN490_c0_g1	KRA_DN48287_c2_g1	1-aminocyclopropane-1-carboxylate oxidase-like	ethylene production, senescence
KOV_DN1335_c0_g1	KRA_DN44770_c0_g1	gibberellin 2-beta-dioxygenase 8-like	GA metabolism
KOV_DN174_c0_g1	KRA_DN60929_c0_g1	serine/threonine-protein kinase SAPK1-like	hyperosmotic stress response
KOV_DN5953_c0_g1	KRA_DN57332_c1_g2	GDSL esterase/lipase 2-like	lipid metabolism
KOV_DN16253_c0_g2	KRA_DN63290_c0_g1	Acetyl-coenzyme A synthetase, mitochondrial	lipid metabolism
KOV_DN33595_c1_g1	KRA_DN54044_c1_g1	caleosin 1	lipid metabolism, stress response
KOV_DN58204_c0_g1	KRA_DN61032_c0_g1	lipase-like PAD4	lipid metabolism, stress response
KOV_DN19808_c0_g2	KRA_DN65272_c0_g1	glutelin type-A 3-like	nutrition
KOV_DN772_c0_g1	KRA_DN58790_c0_g1	protein NRT1/PTR FAMILY 2.9	nutrition, nitrate transport
KOV_DN17288_c1_g1	KRA_DN38866_c0_g1	peroxidase 27-like	oxidative stress response
KOV_DN28823_c0_g1	KRA_DN48528_c1_g2	peroxidase 5-like	oxidative stress response
KOV_DN23154_c0_g1	KRA_DN56053_c0_g3	peroxidase 5-like	oxidative stress response
KOV_DN13196_c0_g1	KRA_DN63168_c0_g1	aldehyde dehydrogenase family 3 member H1-like	oxidative stress response
KOV_DN6075_c0_g1	KRA_DN52711_c1_g1	flavonoid 3′-monooxygenase-like	oxidative stress response, antioxidant synth.
KOV_DN11776_c0_g1	KRA_DN52985_c1_g1	heavy metal-associated isoprenylated plant protein 6	oxidative stress response, heavy metal ass.
KOV_DN3256_c0_g1	KRA_DN64806_c0_g1	protein SRG1-like	oxidative stress response, senescence
KOV_DN8862_c1_g1	KRA_DN71589_c1_g2	protein NRT1/PTR FAMILY 1.2-like	peptide transport
KOV_DN3658_c0_g1	KRA_DN57822_c0_g1	aspartic proteinase PCS1	proteolysis
KOV_DN12803_c0_g1	KRA_DN41740_c0_g1	basic 7S globulin-like	proteolysis, nutrition
KOV_DN16367_c0_g2	KRA_DN47392_c0_g2	Basic 7S globulin	proteolysis, nutrition
KOV_DN1246_c0_g1	KRA_DN45140_c0_g1	F-box protein SKP2A-like	proteolysis, ubiquitination
KOV_DN817_c0_g1	KRA_DN73170_c8_g1	putative ABC transporter C family memb. 15	response to oxidative stress, metabolism of glutathion
KOV_DN3628_c0_g1	KRA_DN72682_c4_g2	alpha-aminoadipic semialdehyde synthase	lysine degradation, stress response
KOV_DN8089_c0_g2	KRA_DN72242_c0_g1	DEAD-box ATP-dependent RNA helicase 14	rRNA processing, ribosome biogenesis
KOV_DN11614_c0_g1	KRA_DN48220_c0_g3	Senescence regulator	senescence
KOV_DN9969_c0_g3	KRA_DN65248_c0_g2	protein DMP3	senescence
KOV_DN15831_c0_g2	KRA_DN53939_c3_g1	cytochrome P450 71A4-like	senescence, flavor production
KOV_DN25654_c0_g1	KRA_DN40946_c0_g1	ribonuclease 1-like	senescence, PI starvation
KOV_DN4090_c0_g1	KRA_DN58419_c0_g3	60 kDa jasmonate-induced protein	stress response
KOV_DN9074_c0_g1	KRA_DN68068_c0_g1	pumilio homolog 5	stress response
KOV_DN20724_c0_g1	KRA_DN59779_c0_g3	bifunctional epoxide hydrolase 2	stress response, cutin biosynthesis
KOV_DN418_c0_g1	KRA_DN46150_c0_g1	auxin-binding protein ABP19a-like	stress response, germin
KOV_DN2633_c0_g2	KRA_DN69004_c2_g1	auxin-binding protein ABP19a-like	stress response, germin
KOV_DN429_c0_g1	KRA_DN35498_c0_g1	alpha-amylase-like	sugar metabolism
KOV_DN1973_c0_g1	KRA_DN44419_c0_g2	cell wall/vacuolar inhibitor of fructosidase 1	sugar metabolism
KOV_DN3558_c0_g1	KRA_DN66372_c0_g1	putative beta-galactosidase	sugar metabolism
KOV_DN2574_c0_g1	KRA_DN59605_c4_g1	beta-D-xylosidase 1	sugar metabolism, cell wall
KOV_DN6104_c0_g1	KRA_DN52927_c0_g4	probable trehalose-phosphate phosphatase D	sugar metabolism, drought stress response
KOV_DN5263_c0_g1	KRA_DN43951_c0_g1	NAC domain-containing protein 92-	TF, abotic stress response
KOV_DN6211_c0_g3	KRA_DN58757_c2_g1	ethylene-responsive transcription factor ABR1	TF, cold stress response
KOV_DN40310_c0_g1	KRA_DN60425_c0_g1	ethylene-responsive transcription factor ERF073	TF, cold stress response
KOV_DN88395_c0_g2	KRA_DN62269_c0_g1	ethylene-responsive transcription factor 2	TF, cold stress response
KOV_DN45283_c0_g1	KRA_DN57848_c2_g2	homeobox-leucine zipper protein ATHB-6	TF, drought stress response
KOV_DN650_c0_g1	KRA_DN58037_c6_g1	zinc finger protein ZAT10-like	TF, drought stress response
KOV_DN2019_c0_g1	KRA_DN59737_c0_g1	homeobox-leucine zipper protein ATHB-12	TF, drought stress response
KOV_DN883_c0_g1	KRA_DN52066_c0_g1	NAC domain-containing protein 72-like	TF, oxidative stress response
KOV_DN3909_c0_g1	KRA_DN53835_c0_g2	B3 domain-containing protein At2g36080-like	TF, repressor
KOV_DN1263_c0_g1	KRA_DN54658_c0_g1	homeobox-leucine zipper protein HAT5	TF, salt stress response
KOV_DN4889_c0_g2	KRA_DN44027_c0_g1	protein CUP-SHAPED COTYLEDON 3	TF, SAM development, organ separation
KOV_DN6423_c0_g2	KRA_DN47072_c0_g3	NAC transcription factor 29-like	TF, senescence
KOV_DN3487_c0_g1	KRA_DN57553_c3_g2	30S ribosomal protein	translation

TF—transcription factor. Green color—proteins likely targeted to plastids. Blue color—proteins likely targeted to mitochondria.

## Supplementary Materials

The following are available online at https://www.mdpi.com/2073-4409/9/12/2700/s1, Figure S1: The comparison of the assemblies made of various minimum length of trimmed reads of the KRA dataset; Figure S2: The comparison of the assemblies made of various minimum length of trimmed reads of the KOV dataset; Figure S3: The enriched GO categories downregulated in female flower buds of Silene vulgaris; Figure S4: The enriched GO categories upregulated in female flower buds of Silene vulgaris; Table S1: Primer sequences and PCR conditions used in this study; Table S2: GO categories downregulated and upregulated in female flower buds of Silene vulgaris; Table S3: The genes downregulated and upregulated in female flower buds of Silene vulgaris, identified by at least one of four bioinformatic pipelines.

## References

[1] Horn R., Gupta K.J., Colombo N. (2014). Mitochondrion role in molecular basis of cytoplasmic male sterility. Mitochondrion.

[2] Touzet P., Meyer E.H. (2014). Cytoplasmic male sterility and mitochondrial metabolism in plants. Mitochondrion.

[3] Hanson M.R. (2004). Interactions of Mitochondrial and Nuclear Genes That Affect Male Gametophyte Development. Plant Cell.

[4] Olson M.S., McCauley D.E. (2002). Mitochondrial DNA diversity, population structure, and gender association in the gynodioecious plant Silene vulgaris. Evolution.

[5] Dufay M., Champelovier P., Käfer J., Henry J.P., Mousset S., Ab Marais G. (2014). An angiosperm-wide analysis of the gynodioecy–dioecy pathway. Ann. Bot..

[6] Renner S.S. (2014). The relative and absolute frequencies of angiosperm sexual systems: Dioecy, monoecy, gynodioecy, and an updated online database. Am. J. Bot..

[7] Bernasconi G., Antonovics J., Biere A.L., Charlesworth D., Delph L.F., Filatov D.A., Giraud T., Hood M.E., Marais G.A.B., McCauley D.W. (2009). Silene as a model system in ecology and evolution. Heredity.

[8] Štorchová H., Olson M.S. (2004). Comparison between mitochondrial and chloroplast DNA variation in the native range of Silene vulgaris. Mol. Ecol..

[9] Barr C.M., Keller S.R., Ingvarsson P., Sloan D.B., Taylor U.R. (2007). Variation in Mutation Rate and Polymorphism Among Mitochondrial Genes of Silene vulgaris. Mol. Biol. Evol..

[10] Sebasky M.E., Keller S.R., Taylor D.R. (2016). Investigating past range dynamics for a weed of cultivation, Silene vulgaris. Ecol. Evol..

[11] Štorchová H., Müller K., Lau S., Olson M.S. (2012). Mosaic Origins of a Complex Chimeric Mitochondrial Gene in Silene vulgaris. PLoS ONE.

[12] Štorchová H., Stone J.D., Sloan D.B., Abeyawardana O.A.J., Müller K., Walterová J., Pažoutová M. (2018). Homologous recombination changes the context of Cytochrome b transcription in the mitochondrial genome of Silene vulgaris KRA. BMC Genom..

[13] Sloan D.B., Müller K., McCauley D.E., Taylor D.R., Štorchová H. (2012). Intraspecific variation in mitochondrial genome sequence, structure, and gene content inSilene vulgaris, an angiosperm with pervasive cytoplasmic male sterility. New Phytol..

[14] Xie Y., Zhang W., Wang Y., Xu L., Zhu X., Muleke E.M., Liu L. (2016). Comprehensive transcriptome-based characterization of differentially expressed genes involved in microsporogenesis of radish CMS line and its maintainer. Funct. Integr. Genom..

[15] Li C., Zhao Z., Liu Y., Liang B., Guan S., Lan H., Wang J., Lu Y., Cao M. (2017). Comparative transcriptome analysis of isonuclear-alloplasmic lines unmask key transcription factor genes and metabolic pathways involved in sterility of maize CMS-C. PeerJ.

[16] Pei X., Jing Z., Tang Z., Zhu Y. (2017). Comparative transcriptome analysis provides insight into differentially expressed genes related to cytoplasmic male sterility in broccoli (Brassica oleracea var. italica). Sci. Hortic..

[17] Chen G., Ye X., Zhang S., Zhu S., Yuan L., Hou J., Wang C. (2018). Comparative Transcriptome Analysis between Fertile and CMS Flower Buds in Wucai (Brassica campestris L.). BMC Genom..

[18] Hamid R., Tomar R.S., Marashi H., Shafaroudi S.M., Golakiya B., Mohsenpour M. (2018). Transcriptome profiling and cataloging differential gene expression in floral buds of fertile and sterile lines of cotton (*Gossypium hirsutum* L.). Gene.

[19] Liu B., Ou C., Chen S., Cao Q., Zhao Z., Miao Z., Kong X., Zhuang F.-Y. (2019). Differentially Expressed Genes between Carrot Petaloid Cytoplasmic Male Sterile and Maintainer during Floral Development. Sci. Rep..

[20] Janzen G.M., Wang L., Hufford M.B. (2018). The extent of adaptive wild introgression in crops. New Phytol..

[21] Haas M., Himmelbach A., Mascher M. (2020). The contribution of cis- and trans-acting variants to gene regulation in wild and domesticated barley under cold stress and control conditions. J. Exp. Bot..

[22] Case A.L., Willis J.H. (2008). Hybrid male sterility in *Mimulus* (Phrymaceae) is associated with a geographically restricted mitochondrial rearrangeMENT. Evolution.

[23] Yamamoto M.P., Shinada H., Onodera Y., Komaki C., Mikami T., Kubo T. (2008). A male sterility-associated mitochondrial protein in wild beets causes pollen disruption in transgenic plants. Plant J..

[24] Stone J.D., Koloušková P., Sloan D.B., Štorchová H. (2017). Non-coding RNA may be associated with cytoplasmic male sterility in Silene vulgaris. J. Exp. Bot..

[25] Krüger M., Abeyawardana O.A.J., Juříček M., Krüger C., Štorchová H. (2019). Variation in plastid genomes in the gynodioecious species Silene vulgaris. BMC Plant Biol..

[26] Song L., Florea L. (2015). Rcorrector: Efficient and accurate error correction for Illumina RNA-seq reads. GigaScience.

[27] Kopylova E., Noé L., Touzet H. (2012). SortMeRNA: Fast and accurate filtering of ribosomal RNAs in metatranscriptomic data. Bioinformatics.

[28] Grabherr M.G., Haas B.J., Yassour M., Levin J.Z., Thompson D.A., Amit I., Adiconis X., Fan L., Raychowdhury R., Zeng Q. (2011). Full-length transcriptome assembly from RNA-Seq data without a reference genome. Nat. Biotechnol..

[29] Li W., Godzik A. (2006). Cd-hit: A fast program for clustering and comparing large sets of protein or nucleotide sequences. Bioinformatics.

[30] Gilbert D.G. (2019). Longest protein, longest transcript or most expression, for accurate gene reconstruction of transcriptomes?. bioRxiv.

[31] Seppey M., Manni M., Zdobnov E.M., Kollmar M. (2019). BUSCO: Assessing genome assembly and annotation completeness. Gene Prediction.

[32] Li B., Fillmore N., Bai Y., Collins M., Thomson J.A., Stewart R., Dewey C.N. (2014). Evaluation of de novo transcriptome assemblies from RNA-Seq data. Genome Biol..

[33] Zhang J., Ruhlman T., Mower J.P., Jansen R. (2013). Comparative analyses of two Geraniaceae transcriptomes using next-generation sequencing. BMC Plant Biol..

[34] Haas B.J., Papanicolaou A., Yassour M., Grabherr M., Blood P.D., Bowden J., Couger M.B., Eccles D., Li B., Lieber M. (2013). De novo transcript sequence reconstruction from RNA-seq using the Trinity platform for reference generation and analysis. Nat. Protoc..

[35] Li B., Dewey C.N. (2011). RSEM: Accurate transcript quantification from RNA-Seq data with or without a reference genome. BMC Bioinform..

[36] Langmead B., Salzberg S.L. (2012). Fast gapped-read alignment with Bowtie 2. Nat. Methods.

[37] Patro R., Duggal G., Love M.I., Irizarry M.I.L.R.A., Kingsford C. (2017). Salmon provides fast and bias-aware quantification of transcript expression. Nat. Methods.

[38] Love M.I., Huber W., Anders S. (2014). Moderated estimation of fold change and dispersion for RNA-seq data with DESeq2. Genome Biol..

[39] Robinson M.D., Oshlack A. (2010). A scaling normalization method for differential expression analysis of RNA-seq data. Genome Biol..

[40] Koloušková P., Stone J.D., Štorchová H. (2017). Evaluation of reference genes for reverse transcription quantitative real-time PCR (RT-qPCR) studies in Silene vulgaris considering the method of cDNA preparation. PLoS ONE.

[41] Stone J.D., Štorchová H. (2014). The application of RNA-seq to the comprehensive analysis of plant mitochondrial transcriptomes. Mol. Genet. Genom..

[42] Sloan D.B., Keller S.R., Berardi A.E., Sanderson B.J., Karpovich J.F., Taylor D.R. (2011). De novo transcriptome assembly and polymorphism detection in the flowering plant Silene vulgaris (Caryophyllaceae). Mol. Ecol. Resour..

[43] Gastaldi V., Lucero L., Ferrero L., Ariel F., Gonzalez D.H. (2020). Class-I TCP Transcription Factors Activate the SAUR63 Gene Subfamily in Gibberellin-Dependent Stamen Filament Elongation. Plant Physiol..

[44] Verma N. (2019). Transcriptional regulation of anther development in Arabidopsis. Gene.

[45] Hu L., Liang W., Yin C., Cui X., Zong J., Wang X., Hu J., Zhang D. (2011). Rice MADS3 Regulates ROS Homeostasis during Late Anther Development. Plant Cell.

[46] Routray P., Li T., Yamasaki A., Yoshinari A., Takano J., Choi W.G., Sams C.E., Roberts D.M. (2018). Nodulin Intrinsic Protein 7;1 Is a Tapetal Boric Acid Channel Involved in Pollen Cell Wall Formation. Plant Physiol..

[47] Cecchetti V., Brunetti P., Napoli N., Fattorini L., Altamura M.M., Costantino P., Cardarelli M. (2015). ABCB1 and ABCB19 auxin transporters have synergistic effects on early and lateArabidopsisanther development. J. Integr. Plant Biol..

[48] Sheng Y., Wang Y., Jiao S., Jin Y., Ji P., Luan F. (2017). Mapping and Preliminary Analysis of ABORTED MICROSPORES (AMS) as the Candidate Gene Underlying the Male Sterility (MS-5) Mutant in Melon (Cucumis melo L.). Front. Plant Sci..

[49] Zluvova J., Zak J., Janousek B., Vyskot B. (2010). Dioecious Silene latifolia plants show sexual dimorphism in the vegetative stage. BMC Plant Biol..

[50] Dobritsa A.A., Geanconteri A., Shrestha J., Carlson A.L., Kooyers N., Coerper D., Urbanczyk-Wochniak E., Bench B.J., Sumner L.W., Swanson R. (2011). A Large-Scale Genetic Screen in Arabidopsis to Identify Genes Involved in Pollen Exine Production. Plant Physiol..

[51] Jiang J., Zhang Z., Cao J. (2012). Pollen wall development: The associated enzymes and metabolic pathways. Plant Biol..

[52] Bosch M., Hepler P.K. (2005). Pectin Methylesterases and Pectin Dynamics in Pollen Tubes. Plant Cell.

[53] Yuan Q., Song C., Gao L., Zhang H., Yang C., Sheng J., Ren J., Chen D., Wang Y. (2018). Transcriptome de novo assembly and analysis of differentially expressed genes related to cytoplasmic male sterility in onion. Plant Physiol. Biochem..

[54] Mittler R. (2002). Oxidative stress, antioxidants and stress tolerance. Trends Plant Sci..

[55] Yang L., Wu Y., Zhang M., Zhang J., Stewart J.M., Xing C., Wu J., Jin S. (2018). Transcriptome, cytological and biochemical analysis of cytoplasmic male sterility and maintainer line in CMS-D8 cotton. Plant Mol. Biol..

[56] Luo D., Xu H., Liu Z., Guo J., Li H., Chen L., Fang C., Zhang Q., Bai M., Yao N. (2013). A detrimental mitochondrial-nuclear interaction causes cytoplasmic male sterility in rice. Nat. Genet..

[57] Yi J., Moon S., Lee Y.-S., Zhu L., Liang W., Zhang D., Jung K.-H., An G. (2016). Defective Tapetum Cell Death 1 (DTC1) Regulates ROS Levels by Binding to Metallothionein during Tapetum Degeneration. Plant Physiol..

[58] Escamez S., Stael S., Vainonen J.P., Willems P., Jin H., Kimura S., Van Breusegem F., Gevaert K., Wrzaczek M., Tuominen H. (2019). Extracellular peptide Kratos restricts cell death during vascular development and stress in Arabidopsis. J. Exp. Bot..

[59] Qin T., Tian Q., Wang G., Xiong L. (2019). LOWER TEMPERATURE 1 Enhances ABA Responses and Plant Drought Tolerance by Modulating the Stability and Localization of C2-Domain ABA-Related Proteins in Arabidopsis. Mol. Plant.

[60] Ding B., Hao M., Mei D., Zaman Q.U., Sang S.-F., Wang H., Wang W.-X., Fu L., Cheng H., Hu Q. (2018). Transcriptome and Hormone Comparison of Three Cytoplasmic Male Sterile Systems in Brassica napus. Int. J. Mol. Sci..

[61] Ilyas M., Rasheed A., Mahmood T. (2016). Functional characterization of germin and germin-like protein genes in various plant species using transgenic approaches. Biotechnol. Lett..

[62] Millar A.H., Whelan J., Soole K.L., Day D.A. (2011). Organization and Regulation of Mitochondrial Respiration in Plants. Annu. Rev. Plant Biol..

[63] Schwarzländer M., König A.-C., Sweetlove L.J., Finkemeier I. (2011). The impact of impaired mitochondrial function on retrograde signalling: A meta-analysis of transcriptomic responses. J. Exp. Bot..

[64] Kühn K., Obata T., Feher K., Bock R., Fernie A.R., Meyer E.H. (2015). Complete Mitochondrial Complex I Deficiency Induces an Up-Regulation of Respiratory Fluxes That Is Abolished by Traces of Functional Complex I. Plant Physiol..

[65] Merendino L., Courtois F., Grübler B., Bastien O., Straetmanns V., Chevalier F., Lerbs-Mache S., Lurin C., Pfannschmidt T. (2020). Retrograde signals from mitochondria reprogramme skoto-morphogenesis in Arabidopsis thaliana via alternative oxidase 1a. Philos. Trans. R. Soc. B Biol. Sci..

[66] Suzuki H., Rodriguez-Uribe L., Xu J., Zhang J. (2013). Transcriptome analysis of cytoplasmic male sterility and restoration in CMS-D8 cotton. Plant Cell Rep..

[67] Niazi A.K., Delannoy E., Iqbal R.K., Mileshina D., Val R., Gabryelska M., Wyszko E., Taconnat L., Szymanski M., Barciszewski J. (2019). Mitochondrial Transcriptome Control and Intercompartment Cross-Talk During Plant Development. Cells.

[68] Zheng B.-B., Wu X.-M., Ge X.-X., Deng X.-X., Grosser J.W., Guo W.-W. (2012). Comparative Transcript Profiling of a Male Sterile Cybrid Pummelo and Its Fertile Type Revealed Altered Gene Expression Related to Flower Development. PLoS ONE.

[69] Hamid R., Jacob F., Marashi H., Rathod V., Tomar R.S. (2020). Uncloaking lncRNA-meditated gene expression as a potential regulator of CMS in cotton (*Gossypium hirsutum* L.). Genomics.

[70] Colombatti F., Gonzalez D.H., Welchen E. (2014). Plant mitochondria under pathogen attack: A sigh of relief or a last breath?. Mitochondrion.

