# Supplementary material for: Differentially Expressed Genes Shared by Two Distinct Cytoplasmic Male Sterility (CMS) Types of Silene vulgaris Suggest the Importance of Oxidative Stress in Pollen Abortion
Source: Cells. 2020 Dec 16;9(12):2700. doi: 10.3390/cells9122700 (PMC7766179; doi:10.3390/cells9122700)
Supplement: Supplementary file 1 [file cells-09-02700-s001.zip › Supplementary figure S1-4 and Table S1.pdf]

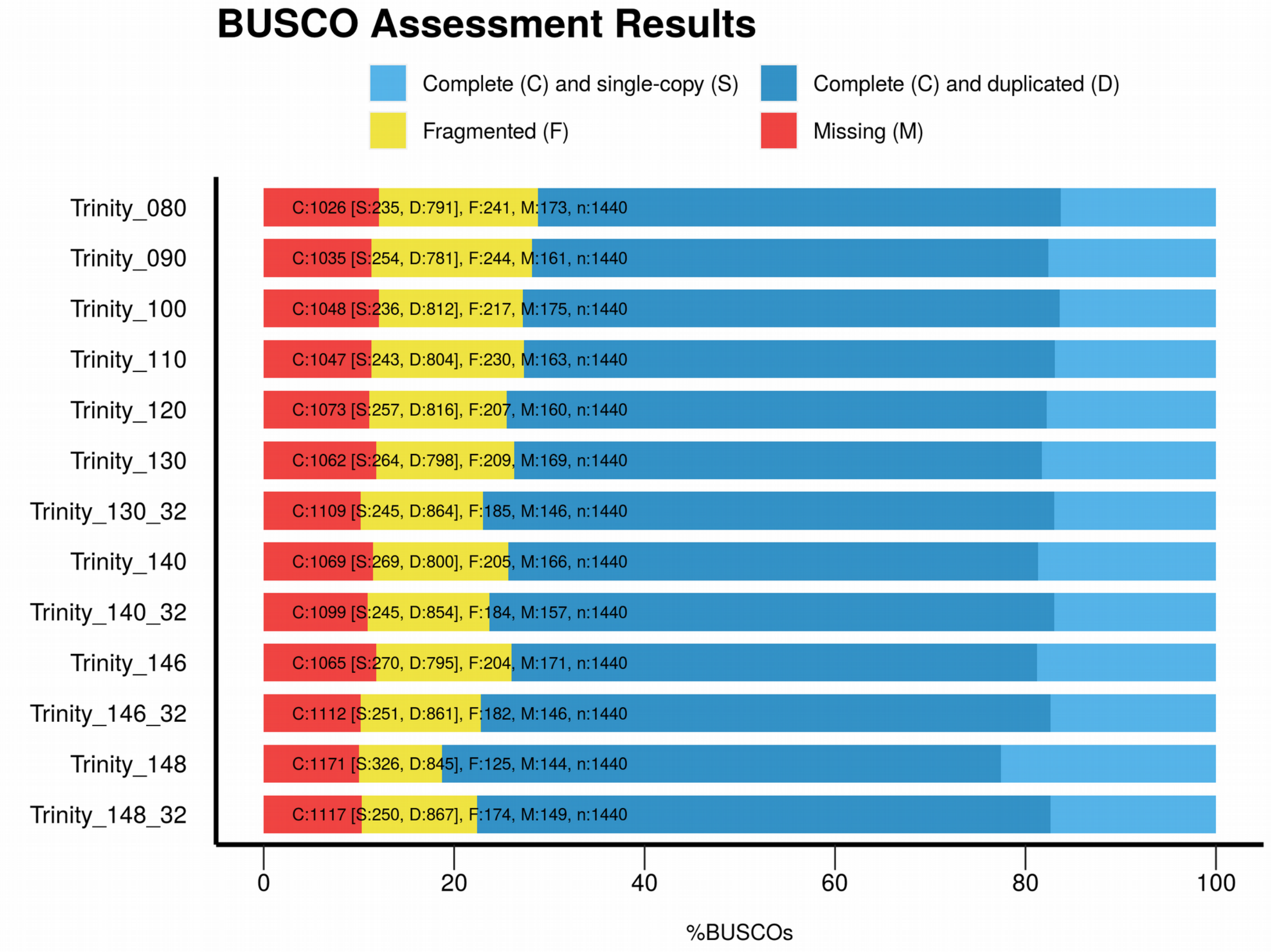

Assembly	Completeness	Contiguity	N50	detonate
Trinity_080	0.905	0.799	2444	-43735258926.12
Trinity_090	0.904	0.798	2437	-43210856665.72
Trinity_100	0.904	0.799	2460	-40618274889.78
Trinity_110	0.905	0.8	2461	-37405947453.29
Trinity_120	0.905	0.805	2469	-33027899925.10
Trinity_130	0.901	0.804	2495	-28546814333.29
Trinity_130_32	0.904	0.824	2484	-43177227963.47
Trinity_140	0.904	0.807	2513	-25854357405.17
Trinity_140_32	0.906	0.822	2520	-40251315557.72
Trinity_146	0.9	0.811	2539	-25058784870.15
Trinity_146_32	0.905	0.827	2528	-39167557352.20
Trinity_148	0.906	0.855	2472	-23210895164.57
Trinity_148_32	0.907	0.831	2542	-36424989540.70

Supplementary Figure 1. The comparison of the assemblies made of various minimum length of trimmed reads of the KRA dataset.

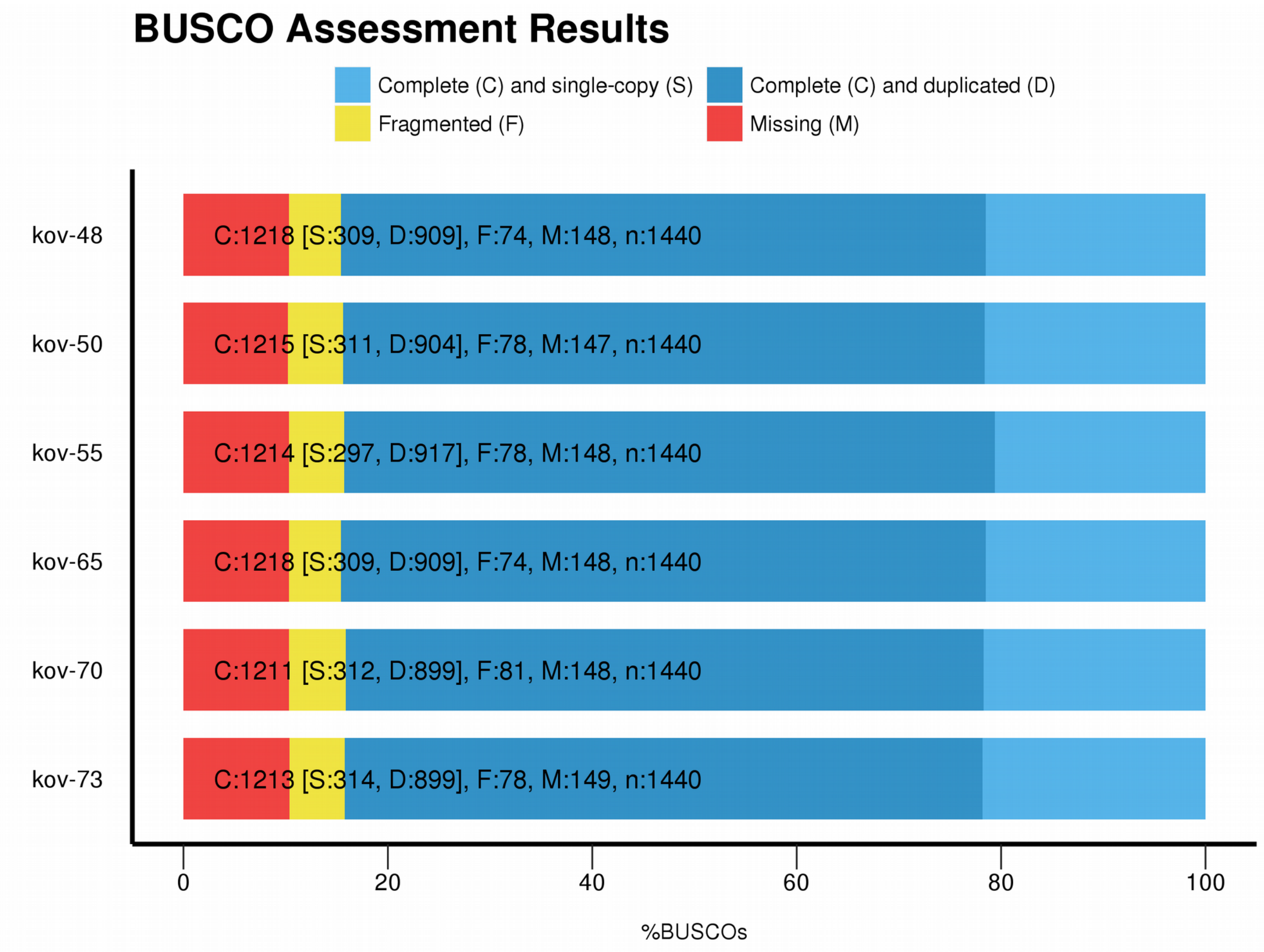

**Assembly**	**Completeness**	**Contiguity**	**N50**	**detonate**
Trinity_kov48	0.91	0.869	1284	-24269497757
Trinity_kov50	0.91	0.866	1278	-24215891686
Trinity_kov55	0.91	0.868	1290	-23938592782
Trinity_kov60	0.91	0.867	1281	-23627447642
Trinity_kov65	0.909	0.868	1280	-23349399191
Trinity_kov70	0.908	0.865	1285	-22536912895
Trinity_kov73	0.908	0.867	1282	-20677574542

Supplementary Figure 2. The comparison of the assemblies made of various minimum length of trimmed reads of the KOV dataset.

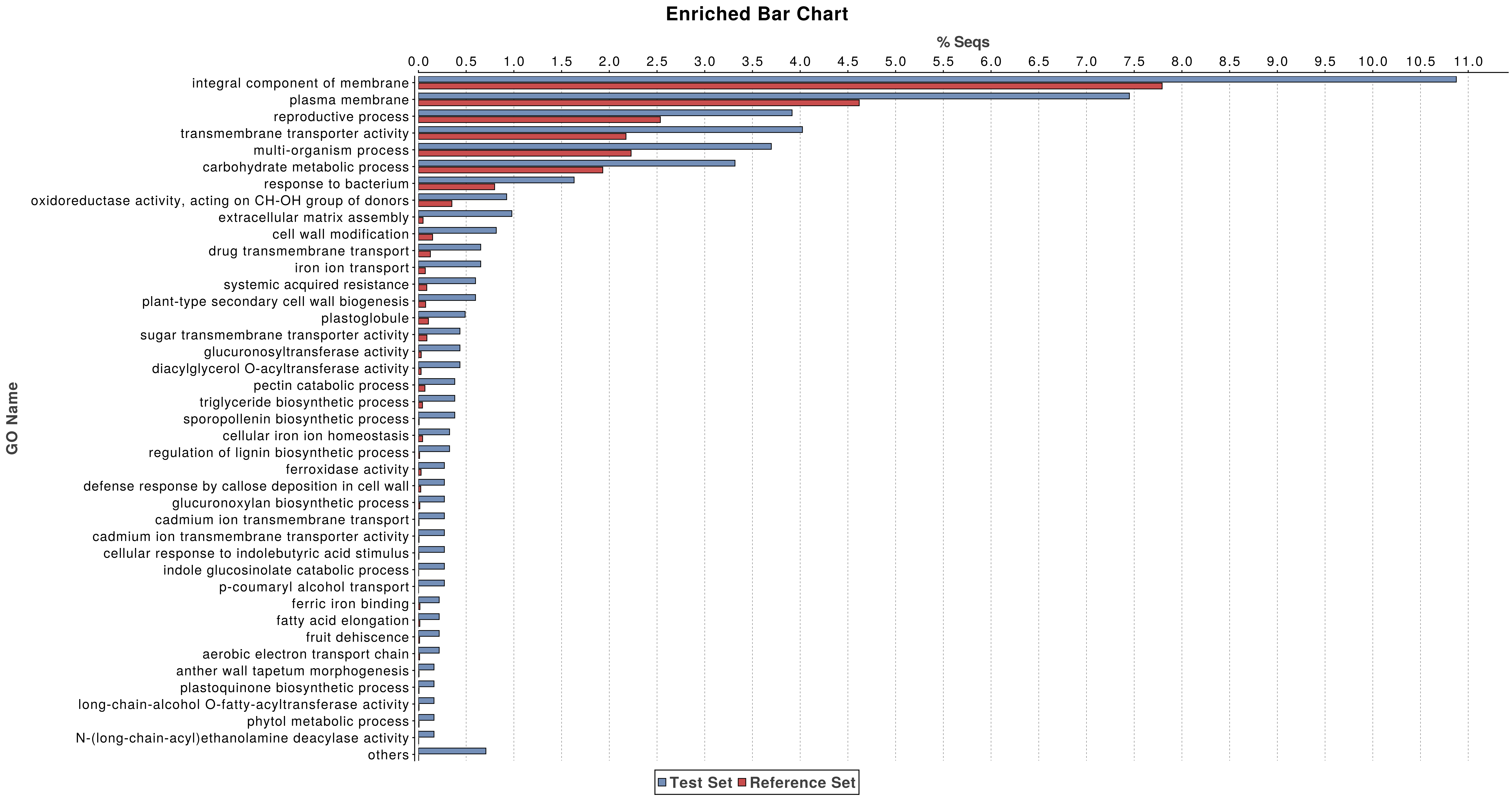

Supplementary Figure 3. The enriched GO categories downregulated in female flower buds of *Silene vulgaris*.

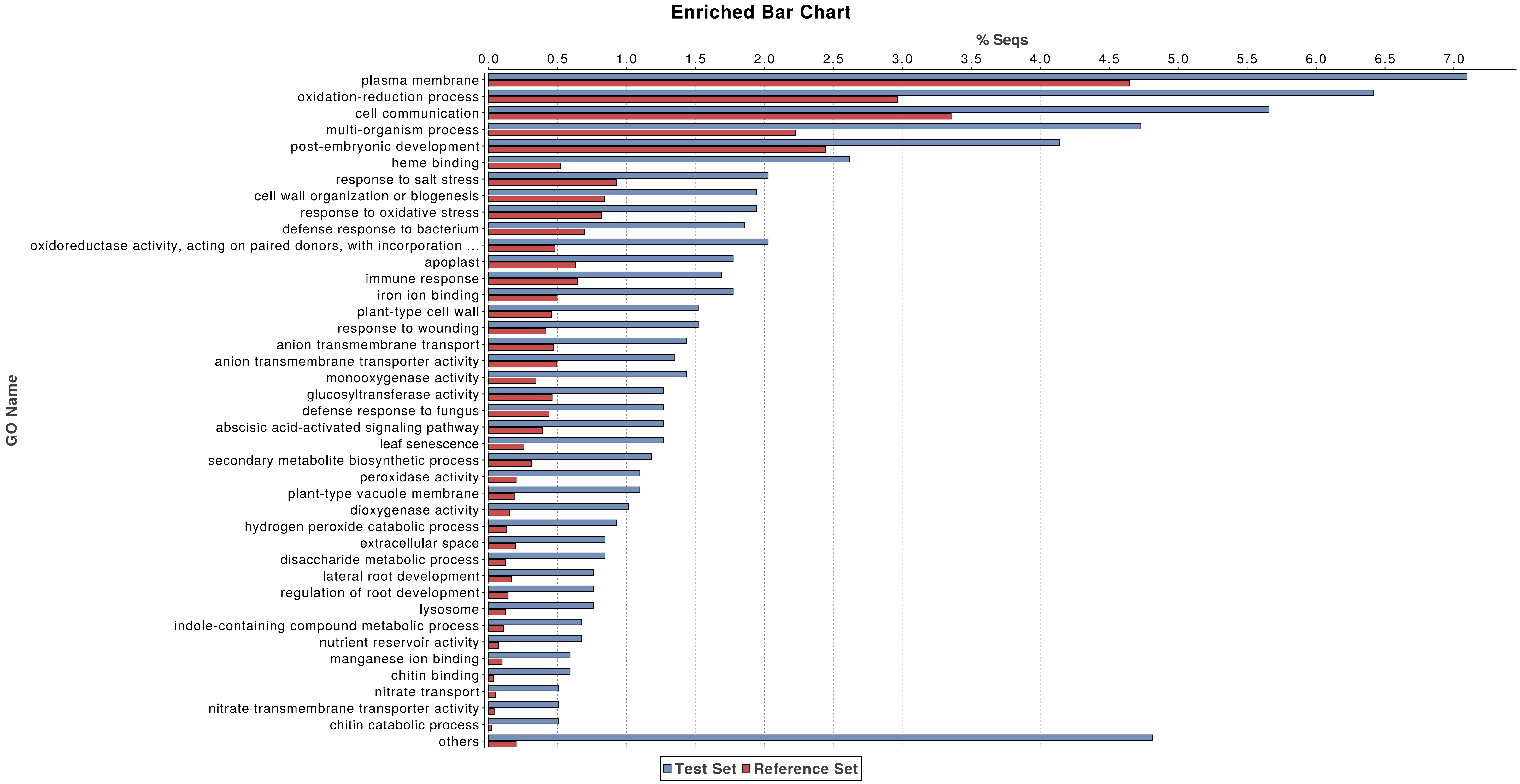

Supplementary Figure 4. The enriched GO categories upregulated in female flower buds of *Silene vulgaris*.

Supplementary Table 1. Primer sequences and PCR conditions used in this study.

**Primers used in RT qPCR**				
Gene	Name	Sequence	Annealing temperature ˚C	Primer concentration µM
SvAOX1	SvAOX1-F2	TATGCCATGGGAGACGTACC	60	0.4
	SvAOX1-R2	GGCATCCATACCTCCTCTGG		
SvPER	PER-F1	CCTGGCCCTAGTTTCAACCTT	62	0.4
	PER-R1	GCGACCAATTGTATGTGCTCC		
SvMC9	Meta-F1	CCGAAACCAACAAATTGGATA	60	0.4
	Meta-R1	AGTTCAGGAAGGCGGAACTTA		
SvERF2	SvERF-F1	GCGGCACTGACTTATGATCG	62	0.4
	SvERF-R1	TTCGGATTGGGTCAGGTTCA		
SvGLP	GLPa-F1	GTGGCTGAGCTCGCTATTCTA	60	0.5
	GLpa-R1	GACTTGAAAATGCGCGAGTGT		
SvCAR4	C2DAR-F1	TGCCCATTCAGCTGACAGTTT	60	0.4
	C2DAR-R1	TGTCGACATGGTTGAACTCTTG		
SvACO4	SvACO4-F1	TGAGTTTCCCTGTGATTAGTTTGG	62	0.4
	SvACO4-R1	TGTTAGCTTCTCCACCGTGTC		
SvACT	SvACT-F	GGGCTGTGATCTCTTTGCTC	60	0.4
	SvACT-R	ATTGTTCGGTATGGAAGCTC		
